# Parental Acculturation and Children’s Bilingual Abilities: A Study With Chinese American and Mexican American Preschool DLLs

**DOI:** 10.3389/fpsyg.2021.761043

**Published:** 2022-01-26

**Authors:** Yuuko Uchikoshi, Mayu Lindblad, Cecilia Plascencia, Helen Tran, Hallie Yu, Krystal Jane Bautista, Qing Zhou

**Affiliations:** ^1^School of Education, University of California, Davis, Davis, CA, United States; ^2^Department of Psychology, University of California, Berkeley, Berkeley, CA, United States

**Keywords:** dual language learners, parental acculturation, preschoolers, oral proficiency, heritage culture, heritage language

## Abstract

Previous studies support the link of parental acculturation to their children’s academic achievement, identity, and family relations. Prior research also suggests that parental language proficiency is associated with children’s vocabulary knowledge. However, few studies have examined the links of parental acculturation to young children’s oral language abilities. As preschool oral language skills have been shown to predict future academic achievement, it is critical to understand the relations between parental acculturation and bilingual abilities with young immigrant children. Furthermore, few studies have examined the links between parental acculturation and children’s bilingual ability among different immigrant groups who live in the same areas to understand possible similarities and differences. To address these gaps, this study examines these relations in two of the largest and fastest-growing immigrant populations in the United States, Chinese American and Mexican American families. A total of 119 dual language learners (DLLs; 64 Chinese Americans and 55 Mexican Americans) enrolled in Head Start programs in Northern California were recruited. DLLs were assessed on oral language measures in both their heritage language (HL) and English. Parental interviews were conducted to obtain parental acculturation and language proficiency. Results showed no significant group differences between Chinese American and Mexican American parents on the majority of their acculturation dimensions. Furthermore, there were no significant group differences in the bilingual abilities between Chinese American and Mexican American DLLs. Cluster analysis identified four groups of DLLs based on their bilingual ability: high language ability in both English and HL, low language ability in both, English-dominant, and HL-dominant. Results suggest that parental acculturation levels are more similar than different among the four groups. On average, parents in all four groups had stronger ties to their heritage culture and HL than to the American culture. Results also showed links between parental cultural identities and children’s language dominance. Parents of English-dominant children had significantly higher levels of American identity than the parents of children with high ability in both languages. Implications are discussed.

## Introduction

One out of three children in the United States comes from a household whose home language is not English (Park et al., 2017). Two-thirds of these households speak Spanish, representing the largest number of dual language learners (DLLs) at 16.1 million children. Chinese is the second most common home language in the United States (Batalova et al., 2021) and the percentage of children who speak Chinese as a home language has increased by approximately 35 percent over the past 7 years (Mitchell, 2020). In sum, national data show that large numbers of Chinese- and Spanish-speaking DLLs are enrolled in American schools.

DLLs from immigrant households are often exposed to multiple cultures and languages in their homes and communities. Through their parents and other adults at home, DLLs are exposed to their heritage language (HL) and their heritage culture (Hughes et al., 2006; Ramírez et al., 2020). At the same time, immigrant parents themselves typically undergo various transitions such as cultural, social, behavioral, and psychological changes coming in contact with a new culture (Schwartz M. et al., 2010; Toro and Nieri, 2018). Parents adopt acculturation strategies that vary by individual factors such as age, education, migration reasons, personal factors, and cultural distance between their heritage culture and host culture (Berry, 1997; Williams et al., 2017). In turn, parental acculturation may influence their children’s cultural identities and language development as parents make conscious and unconscious choices in their children’s upbringing and education (Schwartz S. J. et al., 2010; Liu et al., 2017). However, most of the previous studies examining the links between immigrant parents’ acculturation and child outcomes have been focused on adolescents (e.g., Costigan and Dokis, 2006; Oh and Fuligni, 2010; Kim et al., 2015; Glatz et al., 2021), although with a few exceptions (e.g., Chen et al., 2014; Yu et al., 2018). Moreover, only a few studies have examined the relations between parental acculturation and young DLLs’ bilingual abilities (e.g., Oades-Sese and Li, 2011; Tsai et al., 2012; Troesch et al., 2021).

Furthermore, the heterogeneity of immigrant families needs to be examined with data from different immigrant groups. The present study extends past research by examining the parent acculturation levels of two of the largest and fastest-growing immigrant groups in the United States, namely Chinese American and Mexican American parents, and by investigating the relations between parent acculturation and their preschool children’s oral language abilities in their home language and English.

## Integrative Risk and Resilience Model

Immigrant children are immersed in both their home and host cultures and are often faced with challenges stemming from the different values and beliefs of the two cultures. During their childhood years, children learn to navigate and bridge the two cultures and acquire two languages. The integrative risk and resilience model (Suárez-Orozco et al., 2018) provides a framework to understand the risks and resilience of immigrant children as they adapt to their school life in the host community. Immigrant children are influenced by four levels of context that can serve as risks or resources for adaptation: global forces, political and social contexts of reception, microsystems, and individual levels (Suárez-Orozco et al., 2018).

Of these four levels of context, when children are young, they are mostly influenced by the microsystems that consist of their families, schools, and neighborhoods. Families play a crucial role in the lives of young children and will be the focus of this paper. For immigrant children, the degree of familial acculturation to the host culture may also influence how they navigate the challenges of acquiring the culture and language of the school and community. Differences in parental acculturation to the host country may impact the time parents spend with friends from both the new and the heritage culture as well as their own language proficiency. This may influence the language and cultural opportunities for their children. Higher parental acculturation to the host country may provide their children with more exposure to the language and culture of the host country. At the same time, immigrant families may face risks such as cross-cultural conflicts in the beliefs or ideologies between the two countries. For the children, these conflicts may show up as differences in cultural beliefs, traditions, or language usage between the home environment and school environment. Some children may become stronger in one language over the other, while others may maintain a balance between the two languages. As such, it is important to use a person-centered approach with bilinguals to capture the profiles of oral language abilities.

## Parental Acculturation

Acculturation is one’s cultural adaptation to the new country, whereas enculturation is the maintenance of the native, or the original culture (Berry, 1997). Researchers have noted that acculturation and enculturation are parallel processes and the change occurs with both the heritage culture and the host culture in immigrants (Berry, 1997; Zea et al., 2003). Similar to many studies (e.g., Baker, 2018; Costigan and Dokis, 2006; Kang, 2006), we will use the term “acculturation” to refer to both the process of adapting to the new host country and the process of maintaining ties to the country of origin, or heritage culture.

Researchers have suggested that acculturation can be measured by a cultural orientation that includes the following five dimensions: cultural behavior, cultural knowledge, cultural identity, cultural values, and language (Tsai et al., 2000; Zea et al., 2003). Cultural behavior is shown through individuals’ friendships with people who are from the host or the home culture, their participation in cultural events, and their media choices (Tsai et al., 2000; Zea et al., 2003; Chen and Zhou, 2019). Cultural knowledge is related to how much they know about the historical and political contexts of each culture, as well as their commitment to honoring cultural and social celebrations related to their cultures (Zea et al., 2003). Cultural identity is shown through their affiliation with the community they identify with (Tsai et al., 2000; Zea et al., 2003) as well as generation and geographic life experiences of the family history (Kim and Abreu, 2001; Smokowski et al., 2008). Cultural values relate to how close each individual’s beliefs are aligned with each culture and the pride they have in their culture (Tsai et al., 2000; Zea et al., 2003). Lastly, language is measured in use and proficiency in the HL and the new language, as well as in the maintenance of the HL (Tsai et al., 2000; Zea et al., 2003; Smokowski et al., 2008; Calzada et al., 2009; Chen et al., 2014, 2015; Kim et al., 2015; Baker, 2018; Chen and Zhou, 2019).

These five dimensions of acculturation practices have been measured by self-reported questionnaires (Luo and Wiseman, 2000; Zea et al., 2003; Costigan and Dokis, 2006; Calzada et al., 2009; Oh and Fuligni, 2010; Park et al., 2012; Tsai et al., 2012) or through interviews (Curdt-Christiansen, 2009; Kim, 2011). Cultural orientation may differ depending on a variety of factors including social class, reasons for immigration, how long the parent has been in the host country, how much support there is in the neighborhood and surrounding communities, and access to resources (Zhang, 2010). Past research has shown a wide range of acculturation levels for immigrant families (e.g., Oades-Sese and Li, 2011; Shen et al., 2016). Although there is variation in acculturation levels, studies with Chinese immigrant families in the United States have found that the majority of parents held on to their HL and culture because HL was the only communication tool for parents who had limited English proficiency (Zhang, 2010). Additionally, studies with Mexican American families have found that HL and culture maintenance were protective factors for Latinos (Escobar et al., 2000).

Most of the studies on the effects of parental acculturation on immigrant children have been conducted with parents of adolescents (Costigan and Dokis, 2006; Oh and Fuligni, 2010; Kim et al., 2015) and there is limited research describing the effects of acculturation on preschool-age children (e.g., Oades-Sese and Li, 2011). In particular, there are only a few studies that have examined how parental acculturation attitudes are related to children’s bilingual ability (Tsai et al., 2012; Troesch et al., 2021). Findings from these studies are mixed. One study with immigrant families in Switzerland found no relation between parental acculturation attitudes toward the host country and children’s second language (L2) skills, but found that parental acculturation attitudes toward the country of origin were negatively correlated with children’s L2 skills (Troesch et al., 2021). This is in contrast to a study with Chinese immigrant families that found no connection between parental acculturation orientation toward host and heritage cultures and children’s L2 skills (Tsai et al., 2012). However, this study found a positive association between parents’ Chinese orientation and children’s Chinese expressive language ability via parents’ Chinese language use (Tsai et al., 2012). Another study with Latino families found that children whose parents had acculturated more to the American culture had higher levels of English compared to children whose parents had low acculturation styles (Oades-Sese and Li, 2011). At the same time, children from less acculturated families had higher Spanish language skills than their peers from more acculturated families (Oades-Sese and Li, 2011).

More studies are needed that examine all the dimensions of parental cultural orientation in one study. Moreover, immigrant groups are heterogenous and more studies need to be conducted with different immigrant groups.

## Links Between Parental Acculturation and Children’s Bilingual Abilities

Passing on the heritage culture and HL to children are culturally salient socialization tasks for immigrant parents. Numerous studies have shown socioemotional and cognitive benefits in maintaining HL for both Chinese and Mexican children (Luo and Wiseman, 2000; Zea et al., 2003; Calzada et al., 2009; Oh and Fuligni, 2010; Park et al., 2012; Tsai et al., 2012). Immigrant parents often expect their children to maintain the HL so that children can communicate with their HL-speaking grandparents (Park and Sarkar, 2007). Further, the maintenance of HL helps children develop their identities where they may be minorities in the community, and the HL becomes a tool to learn about their heritage culture (Curdt-Christiansen, 2009). Moreover, HL ability and ethnic identity can contribute to positive parent-child relationships for adolescents and their parents (Oh and Fuligni, 2010). Consistent with these perspectives, parental attitudes toward HL and HL usage have been shown to predict DLLs’ language abilities, particularly DLLs’ HL maintenance among adolescents (Luo and Wiseman, 2000) and preschoolers (Quiroz et al., 2010; Park et al., 2012). Importantly, Park et al. (2012) found the positive relations between parental support of the HL and children’s HL use to be bidirectional. When studying the links between parental acculturation and DLLs’ oral language ability, it is important to assess DLLs’ abilities in both HL and English language. Past research on DLLs’ oral language ability in both languages has been limited compared to the studies with monolingual English-speaking children, mainly due to a lack of assessment instruments that are standardized on DLL populations (Hammer et al., 2014; Chernoff et al., 2021). Many studies on bilingual abilities have been with Spanish-English DLLs (Hammer et al., 2014). In general, studies show that DLLs have smaller vocabularies in each language when compared to monolinguals, but their conceptual vocabularies may be similar to their monolingual age-matched peers (Hammer et al., 2014). As such, in the present study, we examined DLLs’ HL and English language abilities.

Furthermore, to capture individual differences in DLLs’ oral language abilities, a continuous approach (i.e., assessing DLLs’ HL and English language abilities as continuous variables) is preferred over a categorical approach (i.e., comparing bilinguals or DLLs with monolinguals) (Luk and Bialystok, 2013). Some bilinguals are balanced in their abilities in the two languages, while others may be unbalanced (Laketa et al., 2021). The abilities in the two languages may depend on a variety of factors including bilingualism onset, language development, as well as language usage (Laketa et al., 2021). Especially with young DLLs, it is important to capture these varying degrees of bilingual ability as they may change during their childhood years.

## Present Study

Because immigrant families from various cultures seem to have unique acculturation processes, the current study investigates acculturation of the two of the largest immigrant populations in the United States (Park et al., 2017), Chinese American and Mexican American immigrant parents. First-generation immigrant Chinese American or Mexican American parents whose children were attending Head Start programs in Northern California were recruited. The comparison between parents from the two of the largest American immigrant populations (Park et al., 2017) will allow us to examine in what ways these parents are similar and different.

Preschoolers’ oral language skills were the focus of this study as early oral language ability is a key factor in evaluating school readiness and predicts later academic achievement (Snow et al., 1998; Hammer et al., 2014; Mesa et al., 2019). Moreover, we need a better understanding of preschool DLLs’ oral language ability in both languages as they enter early education settings.

Specifically, this study examines the following four research questions.

1. What are the levels of acculturation for first-generation Chinese American and Mexican American parents who have preschoolers enrolled in Head Start programs?
2. What are the levels of oral language abilities in English and HL for children of first-generation Chinese American and Mexican American parents?
3. What are the profiles/clusters of DLLs’ oral language abilities in English and HL?
4. Are parental acculturation dimensions associated with DLLs’ bilingual ability clusters?

In line with previous research, we hypothesized that there will be varying levels of acculturation among the families (Oades-Sese and Li, 2011; Shen et al., 2016). Additionally, we hypothesize that on average DLL children will have low oral language abilities in both English and HL, similar to past studies (Hammer et al., 2014). Previous research suggests that oral language ability levels in HL and English may be similar for Chinese American and Mexican American DLL preschool children (Uchikoshi, 2014). However, we expect variation in children’s oral language ability levels and that there will be several groups based on DLLs’ degrees of bilingualism (Leung and Uchikoshi, 2012; Uchikoshi and Marinova-Todd, 2012). Parental acculturation dimensions may differ according to these bilingual groups. For example, we hypothesize that children with higher levels of oral English language ability may have parents with higher English proficiency than children with lower oral English language ability (Liu et al., 2009; Oades-Sese and Li, 2011).

## Materials and Methods

### Participants

A total of 119 DLLs (64 Chinese American, 55 Mexican American) between 3 and 5.25 years old and their parents were recruited for the study. The Chinese group had 42 boys and 22 girls, and the Mexican group had 24 boys and 31 girls. All participants were recruited from Head Start centers in Northern California, indicating that all are from families with incomes below the poverty guidelines. A maximum of three children per classroom was recruited. The Chinese American children were enrolled in a total of 49 classrooms, while the Mexican American children were enrolled in a total of 37 classrooms. Participation criteria included that both parents identified themselves as Chinese American or Mexican American, their home language was either Chinese (Cantonese or Mandarin) or Spanish, and the children could speak two-word phrases in their HL by age three with no developmental disorders. Both parents and children were compensated for their time at the end of each session. Table 1 presents the demographic information of the preschool DLLs and their primary caregiver.

**TABLE 1:** Descriptive statistics of demographic information.

Variable	Chinese American			Mexican American			
	*Mean*	*SD*	*Range*	*Mean*	*SD*	*Range*	*t*-test
Child Age	3.90	0.64	3–5	4.09	0.57	3–5.25	ns
Parental Age	36.58	4.70	26–46	33.56	6.04	24–45	−2.94***
Parental Education	3.84	1.30	1–7	3.02	1.49	1–6	−3.16***
Parental Years in the United States	10.62	6.92	3–32	17.09	6.66	4–31	4.35***
	*Percentage*			*Percentage*			
Child Born in the United States	92%			89%			

*Parental Education; 1 = less than high school, 2 = some high school, 3 = high school graduate, GED, 4 = technical school, vocational school, certification, some college, associate’s degree, 5 = Bachelor’s degree, 6 = Master’s degree, 7 = Doctorate degree (JD, MD, Ph.D.), ns = not significant.*

***p < 0.001.

Of 119 parents, all were biological mothers except four biological fathers and one adoptive mother. Although Chinese American mothers were significantly older than Mexican American mothers *t* (95) = −2.94, *p* = 0.001, on average the parents in both groups were in their mid-30s. Additionally, although Chinese American parents had significantly more education than Mexican American parents, *t* (104) = −3.16, *p* = 0.001, on average, both groups had a high school diploma. On average, Mexican American parents had lived in the United States significantly longer than Chinese American parents, *t* (66) = 4.35, *p* > 0.001. A total of 59 out of 64 Chinese American children and 49 out of 55 Mexican American children were born in the United States. Four Mexican American children’s information on birthplace was missing.

### Materials and Procedures

This study is part of a longitudinal study and only the data applicable to this study will be discussed. Informed parental consent was obtained in their preferred language at the beginning of the data collection. A team of three trained bilingual research assistants visited the families’ homes to collect data at the most convenient time for the family. The total time varied from 2 to 3 hours, and all data were collected in 1 day. Children were assessed in their dominant language first by bilingual research assistants, followed by their non-dominant language with another bilingual research assistant. Only one language was consistently spoken by the same research assistant to measure the target language. When available, both parents were interviewed to collect data on parental acculturation, but interviews from only the parent who is the main caregiver will be discussed in this paper.

#### Parental Acculturation Measures

Parents were interviewed by bilingual Chinese-English or Spanish-English trained undergraduate research students. All interviews asked parental acculturation questions adapted from the Cultural and Social Acculturation Scale (CSAS; Chen and Lee, 1996), the Familial Ethnic Socialization Measure (FESM; Umaña-Taylor and Fine, 2001), and the Parent Ethnic Identity (PEI; Zea et al., 2003). The three measures combined examined the five dimensions of acculturation (Tsai et al., 2000; Zea et al., 2003). Background information concerning the parent and child was also collected during the parent interviews.

##### Parent Cultural and Social Acculturation

The adult version of the Cultural and Social Acculturation Scale (CSAS; Chen and Lee, 1996) was adapted to assess parental cultural behavior and language proficiency. Specifically, this scale produced three measures that reflect parental cultural behavior: social connections/friends, media usage, and language proficiency. Social connections was measured with a total of six questions (three per culture) on the numbers of friends, frequency in inviting friends, and frequency of being invited to friends’ homes. These questions were asked on a five-point Likert scale (e.g., 1 = “*almost never*” to 5 = “*more than once a week*”). Media usage was measured with a total of ten questions (five per culture) concerning the frequency of reading newspapers, watching TV, listening to radio and music. These questions were asked on a six-point Likert scale (e.g., 1 = “*almost never*” to 6 = “*almost every day*”). Language proficiency was measured with eight questions (four per language) where parents self-rated their language proficiency in reading, writing, speaking, and understanding of spoken language on a four-point Likert scale (e.g., 1 = “*not at all*” to 4 = “*very well*”).

The CSAS has been used in multiple studies with Chinese immigrants (Chen et al., 2014, 2015; Chen and Zhou, 2019) and European Americans (Garrett-Peters and Fox, 2007). The alpha reliabilities for the American social connection, English language proficiency, and English media use for the Chinese American group were 0.77, 0.97, and 0.79, and for the Mexican American group were 0.83, 0.96, and 0.81, respectively. The alpha reliabilities for the heritage social connection, HL proficiency, and HL media use for the Chinese American group were 0.63, 0.93, and 0.66, and for the Mexican American group were 0.66, 0.93, and 0.68 respectively. The reliability for the heritage social connection and HL media scales was under 0.70 for both groups due to the low number of questions to test these aspects. However, as above 0.60 is considered acceptable in the social sciences (Griethuijsen et al., 2014; Taber, 2018), we kept these aspects.

##### Parent Family Ethnic Socialization

The Familial Ethnic Socialization Measure (FESM; Umaña-Taylor and Fine, 2001; Umaña-Taylor et al., 2013) was used to assess parental cultural knowledge and cultural values. This measure consists of 15 questions regarding their values, beliefs, art and music, food, or media on a five-point Likert scale (e.g., 1 = “*not at all*” to 5 = “*very much*”). The Family Ethnic Socialization Measure (FESM) value was created using the average of the 15 questions. Example questions included whether they taught their children about their heritage background, encouraged their children to respect their cultural values and beliefs, participated in activities specific to their heritage culture, and decorated their homes to reflect their cultural backgrounds. Higher scores indicated higher ethnic socialization. The alpha reliabilities for the Chinese American group was 0.92 and for the Mexican American group was 0.91.

##### Parent Ethnic Identity

The Parent Ethnic Identity (PEI) adapted from the cultural identity measure (Zea et al., 2003) was used to measure cultural identity. The measure consists of six questions about American culture and six questions about the heritage culture on a four-point Likert scale (e.g., 1 = “*strongly disagree*” to 4 = “*strongly agree*”). For example, questions asked if they thought of themselves as either American, Chinese/Mexican, whether they were proud to be American, Chinese/Mexican, and how they feel about the cultures. The composite scores for each culture were used. The alpha reliabilities of the American culture for the Chinese American and Mexican American groups were 0.92 and 0.91 respectively. The alpha reliabilities of the heritage culture for the Chinese American and the Mexican American groups were 0.90 and 0.75 respectively.

##### Demographic Measures

Parental age, education, and child’s location of birth were collected along with a variety of other home environment questions adapted from previous surveys (e.g., Uchikoshi, 2014; Chen et al., 2018).

#### Oral Language Ability Measures

Like in previous research with young monolingual and bilingual children (Hammer et al., 2014), children’s oral language skills were measured using the Picture Vocabulary, Oral Comprehension, and Understanding Directions subtests from the Woodcock-Johnson IV Tests of Oral Language (Schrank et al., 2014). Validated parallel measures were available in English and Spanish. For Chinese, we adapted the pictures from the Spanish version as has been done in previous studies (Uchikoshi, 2013; Chung et al., 2019; Chernoff et al., 2021). Both raw and standard scores are reported for English, but only the raw scores are presented for Spanish and Chinese.

##### Picture Vocabulary

This subtest measures expressive oral vocabulary. Children were shown pictures on a page and asked to provide a label for each item verbally. After six consecutive errors, the assessment was discontinued. The procedures were identical for each language. The median test reliability for English at age 4 is 0.94 (McGrew et al., 2014) and for Spanish at age 4 is 0.89 (Wendling et al., 2019). The alpha reliabilities in Chinese for our sample was 0.91.

##### Oral Comprehension

This subtest measures listening, reasoning, and vocabulary skills. In this task, the participants were asked to listen to the audio-recorded passages and supply the missing word using syntactic and semantic cues. The sentences start with simple analogies and progress to complex passages. After six consecutive errors, the assessment was discontinued. The median test reliability coefficient for English at age 4 is 0.86 (McGrew et al., 2014) and for Spanish at age 4 is 0.91 (Schrank et al., 2005). The alpha reliabilities for this sample in Chinese was 0.87.

##### Understanding Directions

This subtest measures children’s receptive oral skills and ability to follow directions. Participants were asked to look at a given image and to point to certain objects on the page. As the participant progressed in the assessment, the prompts became more complex. The assessment was discontinued following the published discontinuation rules. The median test reliability coefficient for English at age 4 is 0.95 (McGrew et al., 2014) and for Spanish at age 4 is 0.93 (Schrank et al., 2005). The alpha reliabilities for this sample in Chinese was 0.90.

## Data Analysis

The study employed descriptive statistics to analyze both parental acculturation levels and DLLs’ oral language skills. Correlations were run to examine relations among the variables. Then, *t*-tests were run to determine significant differences between the Mexican American and Chinese American groups. Cluster analysis was then conducted to identify clusters of children showing similar patterns of bilingual abilities. We then explored possible relations between children’s bilingual ability clusters and parents’ acculturation and enculturation levels.

## Results

The results from the parental interviews, group differences between the Chinese American and Mexican American parental acculturation dimensions, are summarized in Table 2.

**TABLE 2:** Parental acculturation scores by Mexican American and Chinese American.

Variable	Mexican American			Chinese American				
	*Mean*	*SD*	*Range*	*Mean*	*SD*	*Range*	*t*	*p*
CSAS American Friends	1.61	0.72	1.00–4.66	1.84	0.86	1.00–4.00	−1.53	0.13
CSAS Heritage Friends	3.03	0.80	1.33–4.66	2.87	0.70	1.66–4.66	1.15	0.25
CSAS English Proficiency	2.43	0.99	1.00–4.00	2.39	0.73	1.00–4.00	0.20	0.84
CSAS HL Proficiency	3.67	0.55	1.00–4.00	3.46	0.66	1.50–4.00	1.90	0.06
CSAS English Media	2.61	1.28	1.00–5.20	2.66	1.14	1.00–6.00	−0.23	0.82
CSAS HL Media	3.75	0.99	1.80–5.40	3.55	1.11	1.00–6.00	1.06	0.29
FES Total	3.62	0.87	1.73–5.00	3.50	0.75	1.60–5.00	0.81	0.42
PEI American	2.12	0.93	1.00–4.00	2.40	0.73	1.00–4.00	−1.80	0.07
PEI Heritage	3.87	0.24	3.00–4.00	3.39	0.49	2.33–4.00	6.99	<0.001

*CSAS, Cultural and Social Acculturation Scale; HL, Heritage Language; FES, Family Ethnic Socialization; PEI, Parent Ethnic Identity.*

To answer the first question concerning levels of acculturation for first-generation parents who have preschoolers enrolled in Head Start programs, as hypothesized there were varying levels of acculturation among the families. Moreover, the results showed no significant difference in all of the parental acculturation measures between the Chinese American and Mexican American groups except for one. The Mexican American parents rated themselves significantly higher on the Parental Ethnic Identity heritage identity scale than the Chinese American parents; *t* (117) = 6.99, *p* < 0.0001. Although both groups identified less with their American identities, on average, Mexican American parents tended to identify more with their HL identity than their Chinese American counterparts. An analysis of covariance (ANCOVA) with parental years in the United States still found a significant difference between the two groups on the Parental Ethnic Identity heritage identity scale. However, an ANCOVA with parental age and years of education as covariates resulted in no group differences between the Chinese American and Mexican American parents.

On average, parents from both groups had more friends from the heritage culture than American friends. When asked about their English proficiency, on average, both groups reported having “*not well”* proficiency. Parents from both groups reported higher HL proficiency than their English proficiency. Additionally, on average, HL media usage appeared to be higher than English media usage. When asked about how much they taught their heritage cultures to their children on a Likert scale of 1 to 5 (1 = “*not at all”* to 5 = “*very much”*), both groups of parents reported in the middle.

Relations among the acculturation variables were examined with correlation analysis and results are shown in Table 3. Results show that several variables were weakly correlated. Only frequency of English media and English proficiency were positively and moderately correlated, *r*(119) = 0.53, *p* < 0.001.

**TABLE 3:** Correlations among the acculturation variables.

Variable	1	2	3	4	5	6	7	8	9
1 PSAS English Friends	–								
2 PSAS HL Friends	0.391**	–							
3 PSAS English Proficiency	0.266**	0.053	–						
4 PSAS HL Proficiency	0.054	0.122	–0.014	–					
5 PSAS English Media	0.356**	0.142	0.527**	–0.076	–				
6 PSAS HL Media	–0.069	0.112	–0.151	0.259**	0.023	–			
7 FES Total	0.162	0.165	0.083	0.249**	0.229*	0.223*	–		
8 PEI American	0.178	0.042	0.405**	−0.252**	0.261**	–0.119	0.017	−	
9 PEI HL	0.062	0.063	0.081	0.112	0.102	–0.020	0.310**	−0.060	−

CSAS, Cultural and Social Acculturation Scale; HL, Heritage Language; FES, Family Ethnic Socialization; PEI, Parent Ethnic Identity. **p < 0.01; *p < 0.05.

### Children’s Bilingual Abilities

To answer the second question concerning levels of oral language ability in English and HL for children of first-generation Chinese American and Mexican American parents, as hypothesized, on average the children had low scores and wide variation in both languages. When comparing the English mean scores with published age-matched norms, the children in both groups, on average, scored approximately 1.5 standard deviations below the mean. There was a large variation in the scores suggesting varying degrees of bilingualism. Additionally, like previous literature (Uchikoshi, 2014) there were no statistically significant differences between Chinese American and Mexican American DLLs’ achievement scores on all measures in both English and HL as shown in Table 4. Moreover, there were no significant differences based on gender.

**TABLE 4:** Child oral language achievement scores by Mexican American and Chinese American.

Variable	Mexican American			Chinese American				
	*Mean*	*SD*	*Range*	*Mean*	*SD*	*Range*	*t*	*p*
**Raw scores**								
English Picture Vocabulary	11.62	5.70	2–22	11.55	6.36	0–28	0.06	0.95
HL Picture Vocabulary	9.80	7.16	0–22	9.55	6.01	0–23	0.21	0.84
English Oral Comprehension	1.44	2.09	0–7	1.32	3.04	0–14	0.27	0.79
HL Oral Comprehension	1.42	2.17	0–8	0.75	1.53	0–7	1.91	0.06
English Understanding Directions	8.08	6.71	0–22	7.26	6.83	0–27	0.65	0.52
HL Understanding Directions	5.47	5.33	0–20	4.65	4.12	0–15	0.92	0.36
**Standard scores**								
English Picture Vocabulary	75.33	20.72	40–118	78.60	18.08	40–127	−0.90	0.37
English Oral Comprehension	72.43	16.57	57–118	72.34	16.28	49–129	0.03	0.98
English Understanding Directions	81.64	19.15	40–125	79.62	19.09	40–114	0.56	0.58

*HL, Heritage Language.*

Relations among the oral language variables were examined with correlation analysis and results are shown in Table 5. Results show that the within language English and HL measures were all positively and moderately correlated. Cross-language English and HL measures for Picture Vocabulary were negatively and weakly correlated *r*(119) = −0.24, *p* = 0.008, while cross-language English and HL measures for Understanding Directions were positively and moderately correlated, *r*(112) = 0.43, *p* < 0.001.

**TABLE 5:** Correlations among the oral language variables.

Variable	1	2	3	4	5	6
1. HL Picture Vocabulary Raw	–					
2. English Picture Vocabulary Raw	−0.244**	–				
3. HL Oral Comprehension Raw	0.602**	0.104	–			
4. English Oral Comprehension Raw	–0.063	0.619**	0.158	–		
5. HL Understanding Directions Raw	0.320**	0.158	0.378**	0.269**	–	
6. English Understanding Directions Raw	–0.127	0.656**	0.132	0.578**	0.432**	–

HL, Heritage Language. **p < 0.01.

### Cluster Analysis

As there were no differences in English or HL scores between the two groups, we combined the data and conducted agglomerative cluster analysis using Ward’s (1963) method on all participants with English and HL raw expressive vocabulary scores using the scores from the Picture Vocabulary subtest. The vocabulary score was chosen since it showed the largest variations among three oral language measures, and previous studies have also used this expressive vocabulary measure as a proxy for oral language ability (Hammer et al., 2014). This method has been known to be an efficient clustering algorithm to examine underlying data structures. The analysis suggested that four clusters would be the appropriate number of clusters to describe this data. An analysis of variance (ANOVA) on the vocabulary measures supported this decision.

To answer the third question concerning the profiles/clusters of DLLs’ oral language ability in English and HL, cluster analysis results revealed four distinct groups: (a) one group of children had higher English vocabulary scores and lower HL vocabulary scores (English dominant, *n* = 51); (b) one group had lower English vocabulary and higher HL vocabulary (HL dominant, *n* = 18); (c) one group had similarly high English and HL vocabulary (high bilingual, *n* = 21); and (d) one group had similarly low English and HL vocabulary (low bilingual, *n* = 29). Table 6 shows the means, standard deviations, and ranges of the scores for vocabulary and parental acculturation scores by cluster groups.

**TABLE 6:** Child oral language achievement scores by clusters.

	Group 1 English Dominant (*N* = 51)		Group 2 High Bilingual (*N* = 21)		Group 3 Low Bilingual (*N* = 29)		Group 4 HL Dominant (*N* = 18)			
	*Mean*	*SD*	*Mean*	*SD*	*Mean*	*SD*	*Mean*	*SD*	*F*	*p*
English Picture Vocabulary	16.25	3.69	14.38	2.84	4.93	2.83	5.78	2.02	106.92	<0.00
HL Picture Vocabulary	4.55	3.43	17.33	2.71	7.97	3.25	17.94	2.67	129.44	<0.00
CSAS American Friends	1.86	0.83	1.80	0.89	1.67	0.81	1.39	0.53	1.68	0.18
CSAS Heritage Friends	2.95	0.65	3.40	0.95	2.77	0.73	2.67	0.63	4.14	0.01
CSAS English Proficiency	2.57	0.99	2.31	0.60	2.36	0.75	2.14	0.94	1.36	0.26
CSAS HL Proficiency	3.38	0.78	3.61	0.45	3.77	0.39	3.66	0.47	2.82	0.04
CSAS English Media	2.87	1.27	2.60	1.35	2.57	1.03	2.13	0.94	1.77	0.16
CSAS HL Media	3.58	1.08	3.62	1.23	3.77	0.99	3.64	0.93	0.20	0.90
FES Total	3.47	0.78	3.50	0.79	3.72	0.90	3.63	0.76	0.74	0.53
PEI American	2.49	0.87	1.89	0.64	2.30	0.80	2.08	0.86	3.10	0.04
PEI Heritage	3.58	0.52	3.61	0.45	3.57	0.42	3.75	0.36	0.65	0.58

*CSAS, Cultural and Social Acculturation Scale; HL, Heritage Language; FES, Family Ethnic Socialization; PEI, Parent Ethnic Identity.*

The proportions of Mexican American and Chinese American DLLs in each of the four clusters were similar. The English dominant group had the most from the Mexican American group (44% of Mexican Americans, 24 DLLs) and from the Chinese American group (42% of Chinese Americans, 27 DLLs). This was followed by the low bilingual group with 11 Mexican American (20%) and 18 Chinese Americans (28%). The high bilingual group had 10 Mexican Americans (18%) and 11 Chinese Americans (17%). Lastly, the HL dominant group had 10 Mexican Americans (18%) and 8 Chinese Americans (13%).

As expected, an ANOVA analysis on the English vocabulary scores yielded significant differences among the four groups, *F*(3, 115) = 106.92, *p* < 0.0001. A *post hoc* Tukey test showed that the English dominant and high bilingual groups had significantly higher levels of English vocabulary than the other two groups. At the same time, ANOVA analysis on the HL vocabulary scores also yielded significant differences among the four groups, *F*(3, 115) = 129.44, *p* < 0.0001. A *post hoc* Tukey test showed that the HL dominant and high bilingual groups had significantly higher levels of HL vocabulary than the other two groups.

Differences among the cluster groups on child variables, such as age and gender were also examined. Only children’s age was significantly different among the four clusters, *F*(3, 115) = 12.70, *p* < 0.001. The mean age of the English dominant group was 49.75 months, the high bilingual group was 52.33 months, the low bilingual group was 42.10 months, and the HL dominant was 46.56 months. A *post hoc* Tukey test showed that the low bilingual group was significantly younger than the English dominant and high bilingual groups. Additionally, the HL dominant group was significantly younger than the high bilingual group.

An analysis of covariance (ANCOVA) was then conducted with children’s age as a covariate. Even after controlling for children’s age, there were significant differences among the four groups for English vocabulary, *F*(3, 114) = 79.90, *p* < 0.001, and for HL vocabulary *F*(3, 114) = 129.06, *p* < 0.001.

### Links Between Parental Acculturation Dimensions and Children’s Bilingual Ability Clusters

To answer the fourth question regarding the association between parental acculturation dimensions and DLLs’ bilingual ability clusters, we examined the parental acculturation measures among the four clusters. On average, as expected for first-generation immigrant parents (Rumbaut, 2004), all groups had more HL-speaking friends than American friends, had higher HL proficiency than English proficiency, viewed more HL media than English media, and had stronger ethnic identity than American identity. Unlike previous research that found parental English proficiency related to children’s English language ability (Liu et al., 2009), parents in all groups, on average, had similar self-rated English proficiency and similar numbers and quality of relationships with American friends. Their self-rated HL ethnic identities and ethnic values and beliefs were also not significantly different. Both English and HL media usage were also similar among the four groups.

There were also some significant differences in the parental acculturation measures among the four clusters. Although there were no differences in the social connections with American friends, ANOVA results on the relations with HL friends yielded significant differences among the four groups, *F*(3, 115) = 4.14, *p* = 0.0079. A *post hoc* Tukey test showed that the parents of the high bilingual group self-reported as having significantly more HL friends than parents from the low bilingual and HL dominant groups. Additionally, ANOVA results on the parental self-reported HL proficiency showed significant differences among the groups, *F*(3, 115) = 2.82, *p* = 0.0421. A *post hoc* Tukey test showed that the parents from the low bilingual group self-reported as having higher HL proficiency than parents from the English dominant group. However, it should be noted that all groups, on average, reported that they had between “*well*” and “*very well*” HL proficiency. Furthermore, ANOVA results on parental American identity also yielded significant differences among the groups, *F*(3, 113) = 3.10, *p* = 0.0296. A *post hoc* Tukey test showed that parents from the English dominant group self-reported as having a higher American identity than the parents from the high bilingual group.

Differences among the four cluster groups on main caregiver variables, such as age, years in the United States, and years of education were also examined but were not significant.

### Discussion

The findings from this study show that on average, first-generation Chinese American and Mexican American families from low-income communities were very similar in their parental acculturation levels and their preschool-age DLL children’s oral language ability levels. Although there was within-group variation in parental cultural behaviors, our results showed that on average, both groups had more friends from the heritage culture than American friends and viewed more HL media than English media. Both groups’ cultural knowledge and values tended to be in the middle, with a slight inclination toward the HL culture. Additionally, both groups reported having insufficient English proficiency and higher HL proficiency than their English proficiency. It is important to note that different from previous research with Chinese and Mexican immigrant families (e.g., Bodovski and Durham, 2010; Kang et al., 2014), we sampled the two immigrant groups from similar geographic areas in urban Northern California, and both groups were recruited from similar low-income communities (e.g., Head Start families), which may explain the similarities in acculturation dimensions. As for cultural identity, the Mexican American parents on average had a higher HL identity than the Chinese American parents, but their identities toward Americans were similar.

Consistent with past studies, children’s oral language ability levels, when measured in English and HL, were lower than published monolingual norms (Hammer et al., 2014). There were no differences between the Chinese American and Mexican American DLL groups, possibly due to similar schooling experience (Head Start programs), age, and family socioeconomic status.

The results from the cluster analysis revealed differences among DLL children. The majority of children (51 out of 119) were placed in the English dominant group and only 15% of the children were HL dominant, suggesting that even in the preschool years, DLL children’s acquisition of English is rapid and strong. The fact that nearly one-fourth of the children were in the low bilingual group is a concern as this group may continue to show language delays or academic difficulties in the future. However, since the children in the low bilingual group were significantly younger than the children in the high bilingual and the English-dominant groups, perhaps the low bilingual group needed more time to gain their language skills.

This is one of the few studies that have examined the relations between preschool children’s oral bilingual ability and parental acculturation. Despite the variation in children’s bilingual abilities, overall, there were more similarities than differences in parental acculturation across the four groups. Parents in all four bilingual groups also had similar numbers of American friends and on average, they rated themselves as having “not well” English proficiency. The four groups also had similar self-rated HL ethnic identities, values, and beliefs. Yet, there were some significant differences among the group’s parental attitudes and beliefs toward friends from the heritage culture, HL proficiency, and their American identity.

The results show that parents of English dominant children tended to have higher American identity than their counterparts in the high bilingual group, suggesting that parental cultural identity may be associated with children’s language dominance. The higher American identity in parents may be associated with parents using more English, reading more English books to their children, and placing more emphasis on American cultural customs and practices (e.g., celebrating American holidays, watching American sports events) in the home. These opportunities may have strengthened their children’s English oral skills. At the same time, the parents in the high bilingual group may have spent their time balancing the two cultures and two languages, resulting in their children having a more balanced language profile. Further research is needed to examine the quality of parent-child interactions as well as longitudinal research to study the direction of this relationship. It should be noted that the self-rated variations in parental HL proficiency and HL social connections may have been due to self-perception as all groups on average rated their HL proficiency and HL social connections higher than their English proficiency and American social connections. Qualitative research interviewing parents on their experiences would be helpful to understand what the differences may suggest.

Interestingly, there were no differences in parental self-reported English proficiency among the four groups. In past research, children with higher parental English proficiency had higher levels of oral English proficiency than children with lower parental English proficiency (Liu et al., 2009; Oades-Sese and Li, 2011). Differences may not have been seen in this study since many parents reported having “*not well”* English proficiency. Furthermore, this may be due to the fact that all children were recruited from Head Start programs where they have been exposed to similar amounts of English in the classrooms. Once children start school, the classroom environment has been shown to be one of the main influences on L2 acquisition (Sawyer et al., 2018).

## Limitations and Future Research

The current study has some limitations that should be mentioned. As the data was collected at one point in time, it is difficult to conclude whether parental acculturation leads to children’s oral language ability or vice versa. Longitudinal studies with DLL children are urgently needed to understand the developmental and educational needs of these children and their families. It is critical for future studies to follow families longitudinally. Additionally, qualitative analysis of parent-child relationships and interactions is needed to further understand the relations between parental acculturation and children’s oral language ability and should be the focus in future research. Moreover, assessments developed for DLL children that integrate language abilities in both their HL and English are greatly needed to help us understand the development of bilingualism in DLL children.

## Conclusion and Implications

Findings from this study highlight the similarities between the Chinese American and Mexican American parents’ acculturation levels and their DLL preschool children’s oral language ability levels. As both parental groups tended to have more friends from their heritage culture than American friends, viewed more HL media than English, and had higher HL proficiency than English, it appears that both immigrant groups have strong cultural ties to their heritage culture and language. Community organizations and support groups may play important roles when communicating with these groups. Moreover, these findings suggest that communications with DLL parents need to be available in the home language. Most parents were not confident in their English skills, recommending the need for documents written in HL as well as bilingual staff or interpreters to help in communication efforts. However, as there was wide variation in the groups, educators and policymakers need to understand the parental acculturation levels and parental desires when developing and implementing instructional strategies and policies. Given parents’ varying acculturation levels, it is crucial that interventions and recommendations account for each family’s cultural values and acculturation levels.

At the same time, as children are developing their oral language skills, it is important that parents realize that exposing children to both HL and English can be beneficial to their children’s bilingual and bicultural development. The findings from this study suggest that parental cultural identity may be related to their children’s bilingual and bicultural development. Parents may need to be encouraged and supported to use the HL with their children so that their children can acquire bilingual abilities, not only English language ability. English skills may be taught and developed in schools, but unless their children are in bilingual classrooms, parents may need to adopt a conscious effort to maintain and develop their children’s HL. Moreover, educators need to emphasize that such HL support is useful and a valuable contribution to children’s language development. This adds to past research findings that encourage the use of the HL to enrich the home language environment which further supports future academic skills (e.g., Tamis-LeMonda et al., 2019). Family engagement practices in the heritage culture while using the heritage language may be key for DLL children to develop their bilingual abilities that may ultimately lead to their academic success.

## Data Availability Statement

The raw data supporting the conclusion of this article will be made available by the authors, without undue reservation.

## Ethics Statement

The studies involving human participants were reviewed and approved by University of California, Berkeley IRB. Written informed consent to participate in this study was provided by the participants’ legal guardian/next of kin.

## Author Contributions

All authors contributed to the conception and design of the study, writing and revision of the manuscript, and approval of the submitted version.

## Conflict of Interest

The authors declare that the research was conducted in the absence of any commercial or financial relationships that could be construed as a potential conflict of interest.

## Publisher’s Note

All claims expressed in this article are solely those of the authors and do not necessarily represent those of their affiliated organizations, or those of the publisher, the editors and the reviewers. Any product that may be evaluated in this article, or claim that may be made by its manufacturer, is not guaranteed or endorsed by the publisher.
